# Potential gross and net N_2_O production by the gut of different termite species are related to the abundance of nitrifier and denitrifier groups

**DOI:** 10.3389/frmbi.2022.1017006

**Published:** 2022-10-18

**Authors:** Edouard Miambi, Thi My Dung Jusselme, Charline Creuzé des Châtelliers, Alain Robert, Abigail Delort, Xavier Le Roux

**Affiliations:** ^1^ Institut d’Ecologie et des Sciences de l’Environnement de Paris (iEES), Université Paris Est Créteil, Créteil, France; ^2^ Laboratoire Eau Environnement et Systèmes Urbains (Leesu), Université Paris Est Créteil, Créteil, France; ^3^ Laboratoire d’Ecologie Microbienne (LEM), UMR INRAE 1418, UMR CNRS 5557, VetAgroSup, Université Lyon 1, Villeurbanne, France

**Keywords:** gross N2O production, N2O reduction, N-cycling functional genes, potential denitrification, termite

## Abstract

Termites can be a significant source of N_2_O emissions in tropical and subtropical ecosystems. The emission rates vary greatly between species, with many species creating emission hotspots while others acting as net sinks. We studied the relationships between net and gross N_2_O production/consumption and the abundances of eight nitrifier and denitrifier groups (as determined by functional marker genes) in termite gut homogenates for eleven species from five feeding guilds. Overall, the abundance of nitrite reducers and, to a lesser extent, nitrifiers in the gut was strongly correlated with gross N_2_O production, whereas N_2_O reduction was correlated with the abundance of *nosZ1* N_2_O reducers and the *nosZ1*/*nirK* ratio. Our results show that the differences in gross and net N_2_O production rates between termite species are primarily due to differences in nitrifier and denitrifier abundances, as well as the ratio of related functional gene marker abundances in the guts. N_2_O production rates were negatively correlated to the ratio of *nosZ* to *nir*. N_2_O production rates by live individuals measured for a subset of termite species were lower in the wood-feeding than in other species. Our results suggest that the differences in functional gene abundances may be associated with feeding guild, although this should be tested on a larger number of termite species.

## Introduction

Nitrous oxide (N_2_O) is one of the most potent greenhouse gases (GHGs) in the atmosphere, with a global warming potential of 265–298 times more than CO_2_ (Forster et al., 2007). It is also the primary cause of ozone layer destruction (Ravishankara et al., 2009; Prather et al., 2015). Terrestrial ecosystems are the main source of N_2_O (Davidson, 1991). Over recent decades, anthropogenic N_2_O emissions have gained attention because of the increased N_2_O concentration in the atmosphere. However, existing national inventories do not provide a complete picture of N_2_O emissions because they omit natural sources (Tian et al., 2020).

Termites are the most abundant soil macrofauna in tropical and subtropical ecosystems (Eggleton et al., 1996) and can be a significant natural source of greenhouse gases (Brauman et al., 1992; Konaté et al., 2003) and in particular N_2_O (Brümmer et al., 2009). The metabolic activity of termite gut microbiota maintains steep oxygen gradients within the gut lumen (Brune et al., 1995), which can help nitrification and denitrification processes to occur simultaneously. These two microbial processes are significant sources of N_2_O emission since N_2_O is a by-product of nitrification and a primary product of denitrification (Conrad, 1996). Pioneering studies have shown that N_2_O is produced in the termite guts of soil-feeding species with dissimilatory nitrate reduction to ammonium (DNRA) being the primary process rather than denitrification (Ngugi et al., 2011; Ngugi and Brune, 2012). Another study reported termite N_2_O consumption by individual wood-feeding termites (Majeed et al., 2012). Finally, net N_2_O emission or termite consumption is reported to depend on the feeding guild; wood-feeding termites are generally N_2_O consumers, whereas soil-feeding and fungus-cultivating termites are N_2_O producers (Brauman et al., 2015).

Termite N_2_O consumption is currently the only known N_2_O sink in animal digestive systems. However, the relationship between the termite gut microbiota and termite N_2_O emission/consumption remains unclear and poorly documented. Only one study (Brauman et al., 2015) has searched for possible relationships between N_2_O production by termites and the abundance of nitrifier and denitrifier microorganisms in their gut, and no correlation was found. Furthermore, the abundances and possible roles of some N-cycling microbial groups in termite guts have yet to be investigated. This is the case for N_2_O reducers harboring the *nosZ2* gene, some of which can reduce N_2_O but not produce it (Sanford et al., 2012; Jones et al., 2013), as well as nitrifiers that perform complete ammonium oxidation (comammox) (Daims et al., 2015; van Kessel et al., 2015).

The objectives of the present study were (i) to measure both potential gross N_2_O production and consumption rates in termite gut homogenates for various termite species to assess whether the differences in net production rates between species are mainly due to the gross production rates or the gross consumption rates; (ii) to compare N_2_O production from live individuals with potential net production in gut homogenates for a subset of five termite species, analyzing how production by live termites primarily reflects the balance of N_2_O production and N_2_O consumption in termite gut; and (iii) to determine whether differences in the abundances of nitrifier and denitrifier groups (assessed by quantitative PCR targeting functional marker genes) can explain potential gross N_2_O production and N_2_O consumption rates in the termite gut.

## Materials and methods

### Termites used for the study

The termites were provided by the French National Research Institute for Sustainable Development (IRD), France Nord. The colonies were collected from tropical and temperate biomes and maintained in a rearing room on a 12 h light/dark cycle at 27°C ± 2°C and 80% relative humidity. Eleven termite species from five feeding guilds were used for the experiment on termite guts, while five termite species were selected for the experiment with live termites (**Supplementary Table 1**). The study included soil-feeders (*Cubitermes speciosus* and *Crenetermes albotarsalis)*, wood-feeders (*Nasutitermes lujae*, *Nasutitermes ephratae*, *Microcerotermes parvus, Reticulitermes flavipes* and *Prorhinotermes canalifrons*), grass-feeders (*Trinervitermes* sp. and *Hodotermopsis sjostedti*), a wood/soil-feeder (*Termes hospes*) and a fungus-cultivating termite (*Macrotermes muelleri*). Of the eleven termite species selected, three (*Reticulitermes flavipes*, *Prorhinotermes califrons, Hodotermopsis sjostedti*) are lower termites, and the remaining are higher termites. Higher termite species lack protistan symbionts in the hindgut (Lo and Eggleton, 2010). Termites were identified based on their morphological characteristics, molecular analyses, or both. Because worker caste predominates in termite colonies, they were used in all experiments.

### Measurements of potential gross and net N_2_O production from termite gut homogenates

Potential gross and net N_2_O production by termite gut microbiota were measured using the acetylene inhibition technique (Patra et al., 2005), with some modifications. Briefly, 24 mL of 1007 DZMS mineral medium was put into a 120 mL glass serum vial sealed with a butyl rubber stopper. Glucose and glutamic acid were added at a final concentration of 0.5 mg-C/mL, and KNO_3_ was added at a final concentration of 3.5 mM to provide additional nitrate. There were four replicates of homogenate for each of the eleven termite species. The guts of 100 to 200 termites (depending on the termite size) were dissected and pooled in 10 mL of Ringer solution (Barbosa et al., 2015) before being homogenized with a Pyrex glass tissue grinder. Afterward, the medium was inoculated with 1 mL of the gut homogenate. The vials were purged three times by removing the headspace gas and refilling with helium to ensure anaerobic conditions. The vials were then incubated at 28°C for 120 min with the headspace gas sampled every 30 min to determine net N_2_O production. After 120 min of incubation, acetylene (C_2_H_2_) was added to the headspace resulting in a 10% C_2_H_2_ partial pressure, and the headspace gas was sampled every 30 min thereafter (from 120 min to 240 min of incubation). Because acetylene prevents reduction of N_2_O to N_2_, N_2_O emission measured after the addition of C_2_H_2_ corresponds to the production of N_2_ + N_2_O (gross N_2_O production). The N_2_O concentration was determined using a gas chromatograph (µGC R3000, Santa Clara, CA, USA).

The potential net N_2_O production was calculated using the linear increase in N_2_O concentration during the first 120 min of anaerobic incubation before the addition of C_2_H_2_. The linear increase in N_2_O concentration during the second 120 min of anaerobic incubation with C_2_H_2_ in the headspace yielded potential gross N_2_O production. For each sample, the potential N_2_O consumption rate was calculated as the difference between the potential gross N_2_O production and the potential net N_2_O production. The potential net-to-gross N_2_O production ratio, which corresponds to the denitrification end-product ratio (N_2_O/(N_2_O + N_2_) was also calculated.

### N_2_O emission rates by live termites

N_2_O production from live individuals was measured in a subset of five termite species, *Macrotermes muelleri*, *Cubitermes speciosus*, *Nasutitermes lujae*, *Trinervitermes* sp., and *Hodotermopsis sjostedti* Individual termites (90 to 150, depending on termite size) were placed in sterile 120 mL serum vials (Wheaton Inc., Millville, USA). The vials were sealed with rubber stoppers and incubated at 28°C in the dark. For 120 min, gas was sampled from the headspace every 30 min and analyzed as described above. Each termite species had three to five replicates.

### Quantification of the abundances of nitrifiers and nitrite/N_2_O reducers in the termite gut homogenates

The abundances of nitrifier and denitrifier microorganisms in the gut homogenate were quantified using N-cycle functional marker genes as targets. For each replicate, 15 mL of termite gut homogenate was sampled and centrifuged at 5100 g for 5 min to form a pellet. The DNA in the microbial pellet was extracted according to the manufacturer’s instructions using the DNeasy Blood & Tissue Kit (Qiagen Groups). DNA concentration was determined using the Quant-iTTM PicoGreen dsDNA Assay Kit (Invitrogen, France). The DNA extracted was stored at -20°C until it was used.

Real-time PCR was used to determine the abundances of the functional genes. AmoA (coding for ammonia monooxygenase) from archaea and bacteria was amplified using the primer sets CrenamoA23f/CrenamoA616r (Tourna et al., 2011) and amoA2F/amoA1R (Rotthauwe et al., 1997) for AOA and AOB, respectively. Standards were linearized plasmids containing a cloned fragment of archaeal *amoA* (54d9 fosmid fragment) and bacterial *amoA* (*Nitrosomonas europaea*, GenBank accession number L08050). The primer set coma-244F/coma-659R and comaB-244F/comaB-659R were used to amplify clade A and clade B comammox, respectively (Pjevac et al., 2017). As standards, linearized plasmids containing cloned sequences from comammox clades A (DQ008369.1) and B (GenBank accession number AJ564438.1) were used. The primer sets nirK876/nirK1040 (Henry et al., 2006) and nirSCd3aF/nirSR3cd (Throbäck et al., 2004; Kandeler et al., 2006) were used to amplify the *nirK* and *nirS* genes (encoding the copper and cd1 NO_2_^-^ reductases, respectively). Linearized plasmids containing a cloned fragment of the *nirK* gene of *Sinorhizobium meliloti* 1021 and the *nirS* gene of *Pseudomonas stutzeri* served as standards. The *nosZ1* and *nosZ2* gene sequences (encoding the N_2_O reductases from two distinct clades of N_2_O reducers) were amplified using the primer sets *nosZ2*F/*nosZ2*R (Henry et al., 2006) and nosZ–II–F/nosZ–II–R (Jones et al., 2013), respectively. Linearized plasmids containing a cloned fragment of the *nosZ1* gene of *Pseudomonas stutzeri* and the *nosZ2* gene of uncultured bacterium clone CJEAb111were used as standards. The abundance of *norB* genes encoding nitric oxide (NO) reductases was not the subject of this study. When NO accumulates in cells, it becomes toxic. Denitrifiers with the genes *nirS* or *nirK*, which produce NO are also known to have the *nor* genes. This explains why the *nirS* or *nirK* genes are commonly used as molecular markers to target the denitrifier community that produces NO and N_2_O. A dilution series of the extracted DNA were used to test for PCR inhibition by co-extracted compounds, and no inhibition was found. The final reaction volume was 20 μl and contained 1 μM of each primer, 1X of Quanti Tect Sybr-Green PCR Master Mix (Qiagen, Courtaboeuf, France), 0.1% of T4 gene protein 32 (Qbiogene, Carlsbad, CA USA) and 12.5 ng of DNA extract or DNA standards with 10^2^ to 10^7^ gene copies µl^-1^. Samples were analyzed in duplicate on a Lightcycler 480 (Roche Diagnostics, Meyland, France). Conditions of the amplification reactions are given in **Supplementary Table 2**. The PGE platform (Microbial Ecology UMR1418, Lyon) and the DTAMB platform (FR BioEEnviS, Lyon) were used for all analyses. The results were expressed as gene copy numbers per gram of equivalent dry weight gut. To determine the dry to fresh weight ratio, three samples of 50 termite guts (i.e., 3 replicates x 11 species = 33 samples) were dried in a 105°C oven overnight, followed by cooling in a desiccator for 30 min. The percentage of moisture was determined by calculating the amount of weight lost.

### Statistical analyses

All the analyses were performed using either R (version R 4.0.3) or Statgraphics (Centurion XVI, Sigma Plus, France). Before analysis, the N functional gene abundance data were log-transformed The Kolmogorov-Smirnov test was used to determine the normality of the data. One-way analysis of variance (one-way ANOVA) and *post hoc* Fisher’s LSD test were used to determine differences in N_2_O gross production, N_2_O net production, N_2_O consumption, and abundances of nitrifiers and denitrifiers among termite species. One-way ANOVA was also used to compare net and gross N_2_O production rates as well as microbial abundances between (i) the wood-feeding and soil/wood-feeding termite species and (ii) species from other feeding guilds (i.e., fungus-growing, grass-feeders, and soil-feeders).

Using Pearson’s product-moment coefficient (r), linear relationships were tested between net N_2_O production, gross N_2_O production, and N_2_O consumption, as well as the abundances of the N-related microbial groups. Furthermore, Pearson’s correlation was used to test linear relationships between N_2_O emissions by live termites and gross and net N_2_O production by termite gut homogenates.

The abundances of the N-related microbial groups in termite guts were classified using principal component analysis (PCA). Permutation tests were used to determine whether the PCA ordination of wood- and soil/wood-feeding termite species differed significantly from those of other guilds.

Backward stepwise regression models were used to identify the main predictors of potential gross and net N_2_O production and N_2_O consumption rates by termite gut homogenates, with the most parsimonious set of predictors chosen from the abundances of AOA, AOB, the sum of AOA, and AOB, comA and comB, the sum of comA and comB, *nirK* and *nirS* nitrite reducers, the sum of *nirK* and *nirS*, *nosZ1* and *nosZ2* N_2_O reducers and the sum of *nosZ1* and *nosZ2*. When two explanatory variables were highly correlated (Pearson’s correlation higher than 0.7 or lower than -0.7), they were excluded (Tabachnick et al., 2007). The adjusted coefficient of determination (R^2^_adj_) and Akaike’s information criterion (AIC) were used to assess model performance (Bozdogan, 1987).

## Results

### Potential gross and net N_2_O rates by gut homogenates and live termites

**Figure 1:**
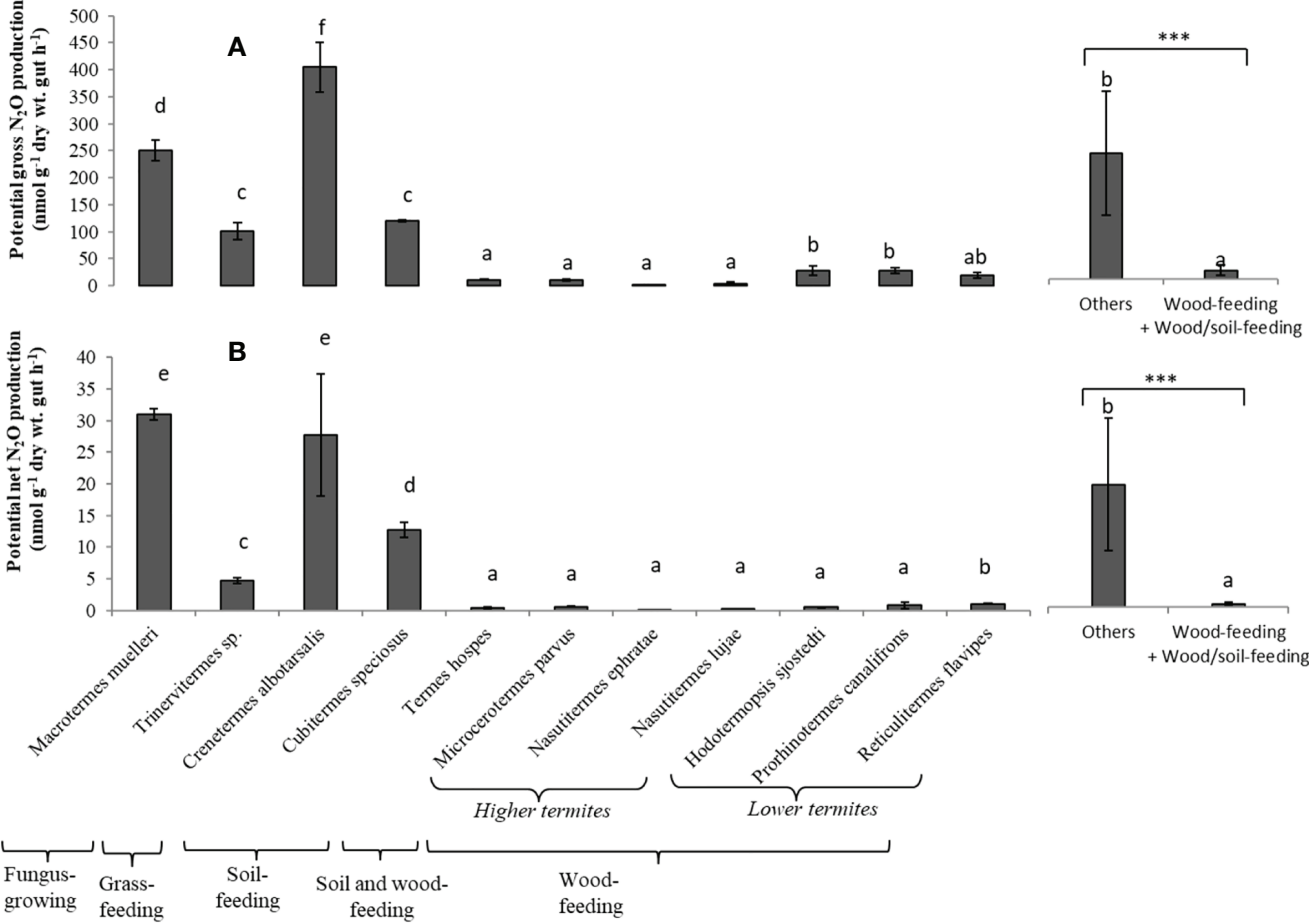
Potential gross N_2_O production **(A)** and potential Net N_2_O production **(B)** from the gut homogenates for each termite species. Different letters indicate significant differences (P < 0.05) between species. Bars indicate the means (and standard deviation) of five replicates. The two bars on the right compare the mean value for wood-feeding and wood/soil-feeding termite species to the value for other species (***p < 0.001, indicating that the mean values are significantly different).

Potential gross N_2_O production rates (i.e., using acetylene which blocks N_2_O reduction) from the termite gut homogenates were about 9 to 22 times higher than potential net production for *Macrotermes muelleri* (fungus-growing termite), *Cubitermes speciosus* and *Crenetermes albotarsalis* (two soil-feeding species) and *Trinervitermes* sp. (grass-feeding termite), with rates ranging from 4.5 to 17.8 µg N-N_2_O h^-1^ (g dry wt. gut)^-1^ (**Figures 1A** **, B**). In contrast, potential gross and net N_2_O production rates by the gut homogenates of the soil/wood-feeding species *Termes hospes* and the six wood-feeding termite species –whether they were higher or lower termites– were low, with rates not exceeding 1.2 µg N-N_2_O h^-1^ (g dry wt. gut)^-1^. Overall, wood-feeding and soil/wood-feeding termite species produced significantly less N_2_O than other species.

**Figure 2:**
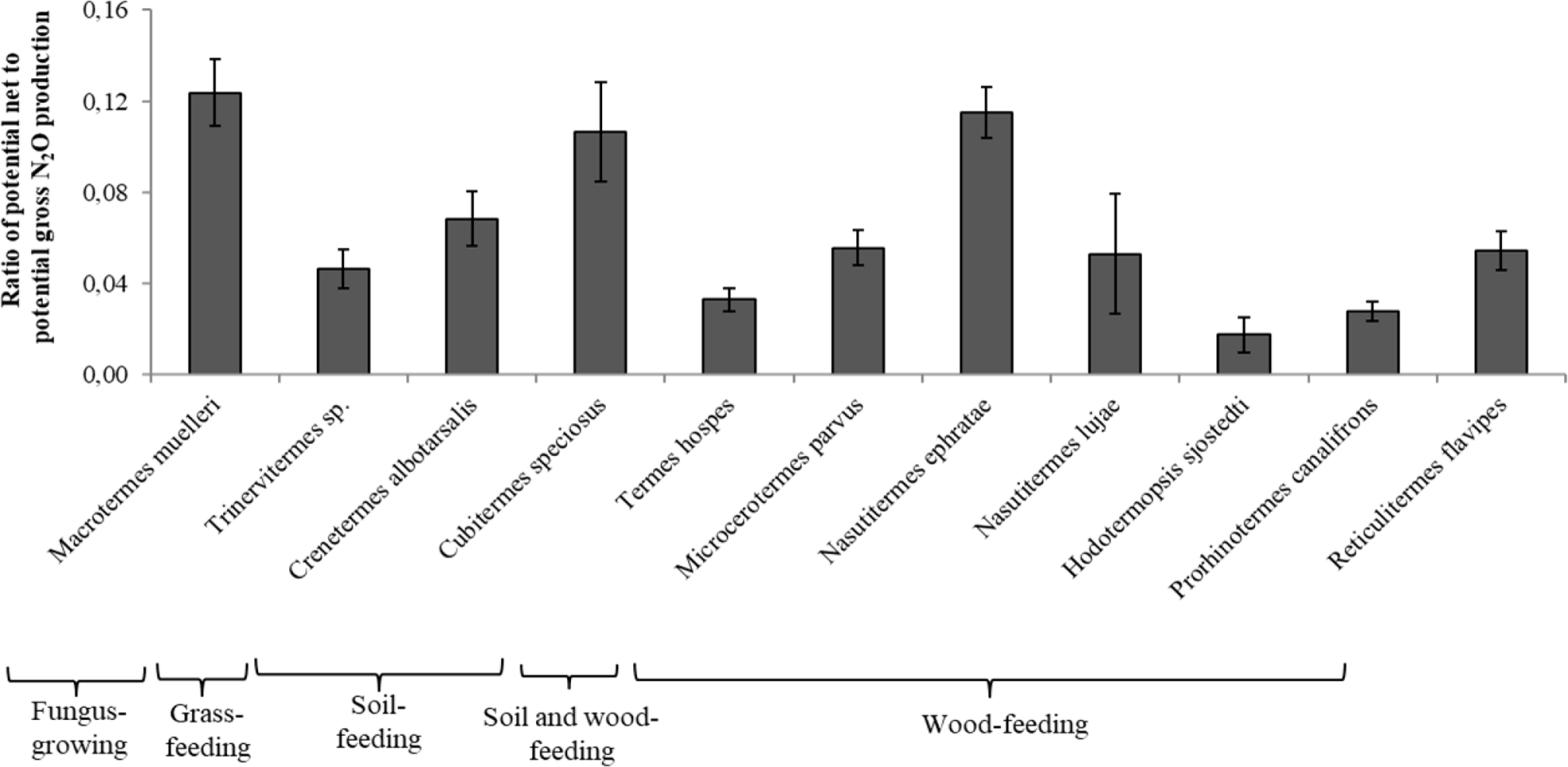
Ratio of potential net N_2_O production to potential gross N_2_O production from the gut homogenates for each termite species. For each ratio, different letters indicate significant differences (P < 0.05) between species. Bars indicate the means (and standard deviation) of five replicates.

The ratio of potential net-to-gross N_2_O production rates was low for the gut homogenates of all the termite species (**Figure 2**). The values were significantly higher for *M. muelleri*, *C. speciosus*, and *N. ephratae*. The lowest ratios were 0.018 and 0.028 for *Hodotermopsis sjostedti, and Prorhinotermes canalifrons* (lower termites), respectively.

**Figure 3:**
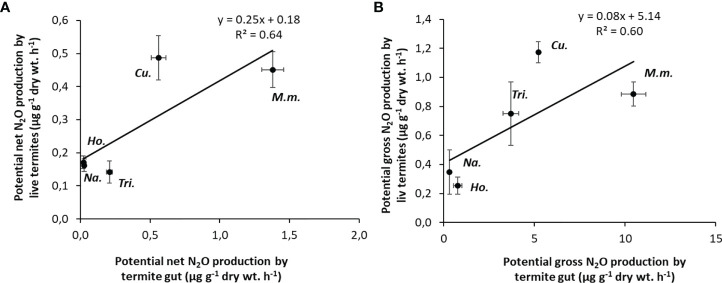
Correlations between potential N_2_O emission rates from live termites and potential N_2_O production rates from the gut homogenates **(A)**; potential gross N_2_O production from live termites and potential gross N_2_O production from gut homogenates **(B)**. Each point corresponds to one species (5 replicates per species). M.m., *Macrotermes mulleri*; Cu., *Cubitermes speciosus*; Tri., *Trinervitermes* sp.; Na., *Nasutitermes ephratae*; Ho., *Hodotermopsis sjostedti*.

N_2_O production rates from live termite individuals were significantly correlated with gross and net N_2_O production rates from the gut homogenates (R^2^ = 0.54 and R^2^ = 0.69, respectively) for a subset of five species (**Figures 3A** **, B**).

### Abundances of nitrifier and denitrifier groups in the termite gut homogenates

**Figure 4:**
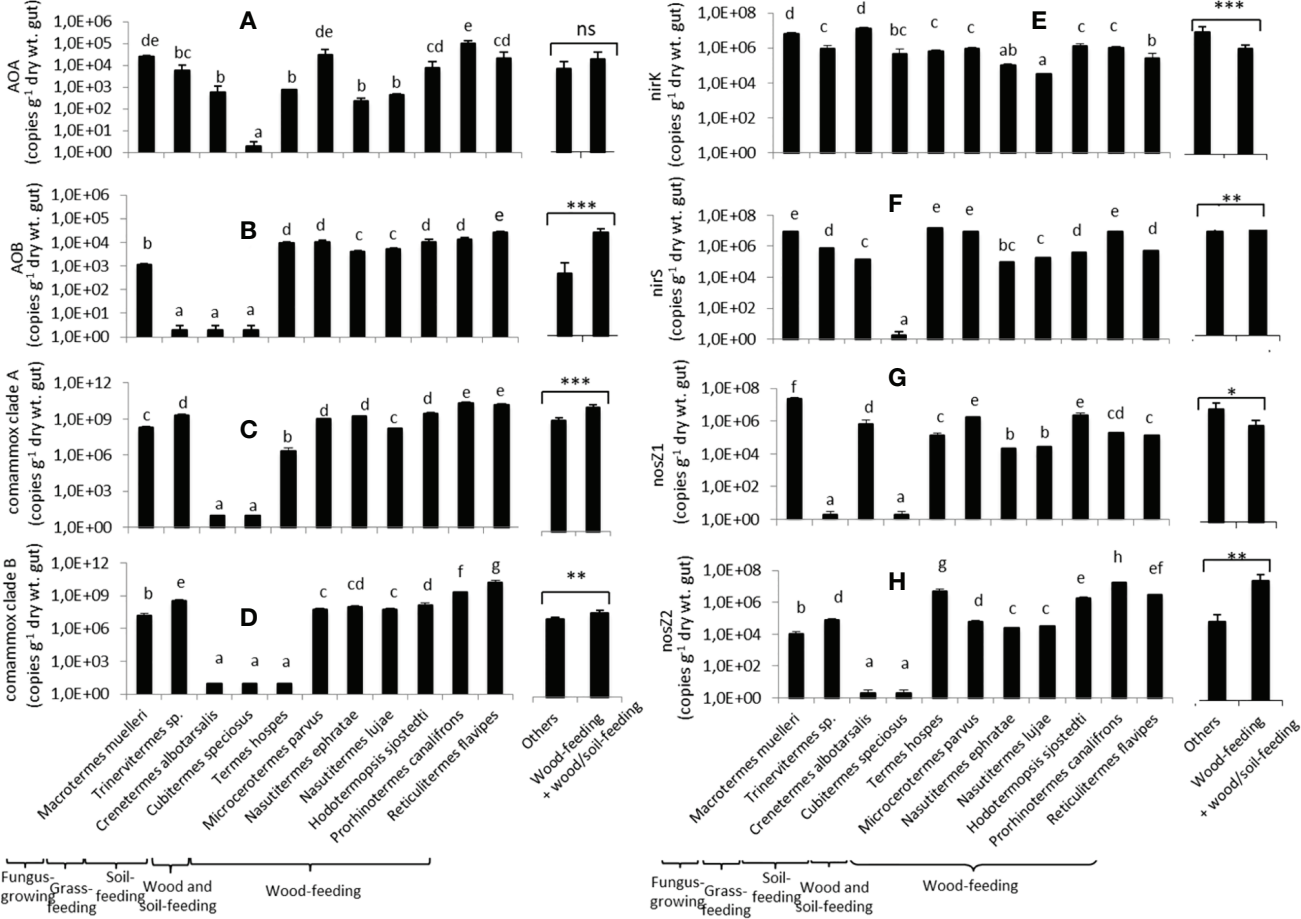
Abundances of the eight nitrifier and denitrifier microbial groups in the gut of the eleven termite species. AOA **(A)** and AOB **(B)** refer to ammonia-oxidizing archaea and bacteria, respectively; comammox clade A **(C)** and clade B **(D)** refer to complete ammonia oxidizers from clades A and B, respectively; *nirK* **(E)** and *nirS* **(F)** refer to *nirK* and *nirS* nitrite reducers, respectively; *nosZ1* **(G)** and *nosZ2* **(H)** refer to *nosZ1* and *nosZ2* N_2_O reducers, respectively. For each gene, different letters indicate significant differences (p< 0.05). The two bars on the right compare the mean abundances for wood-feeding termite species to the mean abundances for the other species (*0.01<p <0.05; **0.01<p<0.001; ***p<0.001; ns: not significantly different).

Ammonia-oxidizing archaea (AOA) abundance in the termite gut homogenates varied according to the termite species (**Figure 4A**), ranging from 10^1^ to 10^5^ copies (g dry wt gut)^-1^. *Cubitermes speciosus* had the lowest values. Ammonia-oxidizing bacteria (AOB) had the same relative abundance as AOA, ranging from 10^1^ to 1.1 x 10^5^ copies (g dry wt gut)^-1^ (**Figure 4B**). AOB abundances, on the other hand, were significantly lower in the grass-feeder *Trinervitermes* sp. and the two soil-feeders (*Cubitermes speciosus*, *Crenetermes albotarsalis).* Except for the two soil-feeding species and *P. canalifrons*, the total abundance of ammonia-oxidizing bacteria was in the same range (**Supplementary Figure 1**). The abundances of comammox clade A and clade B (**Figures 4C** **, D**) were higher than those of canonical nitrifying microorganisms (AOA and AOB) and ranged, from 10^1^ to x 10^10^ copies (g dry wt. gut)^-1^. The abundances of comammox from clades A and B were low in *Cubitermes speciosus* and *Crenetermes albotarsalis*, and clade B abundance was also low in *Termes hospes.* Total comammox abundances were highest in wood-feeding, grass-feeding, and fungus-cultivating termites (**Supplementary Figure 2**).

The abundance of nitrite reducers (harboring the *nirK and nirS* genes) was also species-dependent (**Figures 4E** **, F**). The abundance of *nirK* bacteria ranged from 3.2 x 10^4^ (*N. lujae*) to 1.3 x 10^7^ (*Crenetermes albotarsalis*) copies (g dry wt. gut)^-1^. The abundance of *nirS* bacteria ranged from 10^1^ to 1.5 x 10^7^ copies (g dry wt. gut)^-1^, with *Cubitermes speciosus* having the lowest abundances and for *Termes hospes*, *Microcerotermes parvus, Prorhinotermes canalifrons, Macrotermes muelleri* having the highest Nitrite reducers total abundances (*nirK* + *nirS*) ranged from 2.1 x 10^5^ to 1.6 x 10^7^ copies (g dry wt gut)^-1^ (**Supplementary Figure 3**).

The abundances of N_2_O reducers (harboring the *nos1* and *nosZ2* genes) varied considerably between species (**Figures 4G** **, H**). The abundance of *nosZ1* ranged from 10^1^ to 2.9 x 10^7^ copies (g dry wt. gut)^-1^, with *Trinervitermes* sp. and *Cubitermes speciosus* having the lowest abundances and *Macrotermes muelleri* having the highest. The abundance of *nosZ2* bacteria ranged from 10^1^ to 1.7 x 10^7^ copies (g dry wt. gut)^-1^, with the two soil-feeding species *Crenetermes albotarsalis* and *Cubitermes speciosus* having the lowest abundances and *Prorhinotermes canalifrons* having the highest. The total abundance of N_2_O reducers (**Supplementary Figure 4**) varied greatly as well, ranging from 4.55 x 10^5^ for *Cubitermes speciosus* to 2.49 x 10^7^copies (g dry wt. gut)^-1^ for *M. muelleri*. Overall, the abundances of AOB, comammox clades A and B, *nirS* nitrite reducers, and *nosZ2* N_2_O reducers were higher in wood-feeding and soil/wood-feeding termite species than in other species, while *nirK* and *nosZ1* bacteria were lower in wood-feeding termite species than for the other species.

Except for *Microcerotermes parvus*, most wood-feeding termite species had high ratios of the total abundance of ammonia oxidizers to total abundance of nitrite reducers (*amo*/*nir*, **Table 1**). They also had high ratios of total abundance nitrifiers (ammonia oxidizers and complete ammonia oxidizers) to total nitrite reducer abundance ((*amo+com*)/*nir*). The *nosZ*/*nir* ratio was also higher for wood-feeders than for the soil and grass-feeding species (**Table 1**). Lower termite species (*Hodotermopsis sjostedti*, *Reticulitermes flavipes*, and *Prorhinotermes canalifrons*) had a *nosZ*/*nir* ratio >1 among the wood-feeders,. Comammox *amoA* (clade A and B), *nosZ*2 and AOB *amoA* abundances were also significantly different between higher and lower termites (**Supplementary Table 3**).

**Table 1:** Abundance ratios for ammonia oxidizers-to-nitrite reducers (*amo/nir*), nitrifiers-to-nitrite reducers (*amo*+com/nir), and N_2_O reducers-to-nitrite reducers (*nos/nir*) for the eleven termite species. Values are means ( ± SE) with n=4.

Termite feeding-groups	Termite species	*amo/nir*	*amo+com/nir*	*nos/nir*
Fungus-growing	*Macrotermes muelleri.*	0.002 ± 0.001a	14.4 ± 6.3a	1.64 ± 0.26b
Grass-feeding	*Trinervitermes sp*	0.005 ± 0.005a	144.2 ± 87.4a	0.06 ± 0.01a
Soil-feeding	*Crenetermes albotarsalis *	0.001 ± 0.001a	0.0 ± O.Oa	0.04 ± 0.01a
Soil-feeding	*Cubitermes speciosus*	0.004 ± 0.001a	0.0 ± 0.01a	0.04 ± 0.01a
Soil/wood-feeding	*Termes hospes*	0.001 ± 0.001a	0.1 ± 0.1a	0.31 ± 0.06a
Wood-feeding	*Microcerotermes parvus*	0.004 ± 0.001a	99.1 ± 26.6a	0.18 ± 0.02a
Wood-feeding	*Nasutitermes ephratae*	0.022 ± 0.001b	8764.1 ± 4066.0b	0.23 ± 0.01a
Wood-feeding	*Nasutitermes lujae *	0.035 ± 0.015b	1263.8 ± 793.9a	0.36 ± 0.01a
Wood-feeding	*Hodotermopsis sjostedti*	0.024 ± 0.001b	2284.9 ± 1088.9a	2.73 ± 0.37c
Wood-feeding	*Prorhinotermes canalifrons*	0.008 ± 0.001ab	2472.3 ± 943.3a	1.67 ± 0.03b
Wood-feeding	*Reticulitermes flavipes*	0.010 ± 0.001ab	55800.1 ± 16422c	4.72 + 1.03d

For each ratio, different letters indicate significant differences between species (P < 0.05).

**Figure 5:**
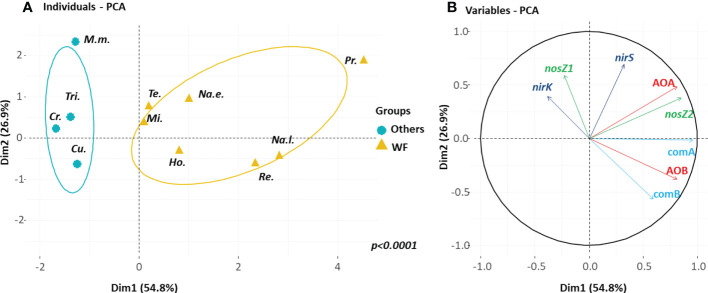
Principal component analysis (PCA) of the N functional gene abundances in the gut of the eleven termite species. **(A)** The plane defined by the two first axes of the PCA distinguishes between wood-and soil/wood-feeding species (WF, yellow brown) and other species (Others, blue). **(B)** The PCA correlation circle where M.m. is *Macrotermes muelleri*, Tri. is *Trinervitermes* sp., Cr. is *Crenetermes albotarsalis*, Cu. is *Cubitermes speciosus*, Te. is *Termes hospes*, Mi. is *Microcerotermes parvus*, Ho. is *Hodotermopsis sjostedti*, Na.e. is *Nasutitermes ephratae*, Na.l. is *Nasutitermes lujae*, Pr. is *Prorhinotermes canalifrons* and Re. is *Reticulitermes flavipes*.

The first two axes of the PCA performed on the gut homogenate samples explained 82% of the total inertia (**Figure 5A**). The wood-feeding termite species and soil/wood-feeding termites differed significantly (p<0.0001) from the other species according to a discriminant analysis carried out with 1,000 permutations. The first axis separated wood-feeding termite species with high abundances of all nitrifier groups (AOA and AOB as well as comammox clade A and B) and *nosZ2* bacteria from other termite species with low abundances of *nosZ1* and *nirK* bacteria (**Figure 5B**).

### Main microbial drivers of gross and net N_2_O production rates

The models relating N_2_O production and consumption to the abundances of the functional genes explained 92%, 78%, and 82% of the variance in gross N_2_O production, N_2_O consumption, and net N_2_O production, respectively (**Table 2**). The total abundance of nitrite reducers (*nir* total; 57% of the variance explained) and, to a lesser extent, the abundance of AOB (an additional 28%) and total comammox (from clades 1 and 2; 12%) were the main drivers of gross N_2_O production. N_2_O consumption was primarily determined by the *nosZ1*/*nirK* ratio (65%), the abundance of *nosZ1* bacteria (24%), and, to a lesser extent, the abundance of *nosZ2* bacteria (11%), with N_2_O reducers having a positive effect on N_2_O consumption. The main drivers that controlled the net N_2_O production were the same as those that controlled net N_2_O consumption, with the *nosZ1*/*nirK* ratio (49%) and *nosZ1* abundance (44%) and, to a lesser extent, the *nosZ2* abundance (7%), and N_2_O reducer abundance having a negative effect on net N_2_O production.

**Table 2:** Best fit models predicting gross N_2_O production, N_2_O consumption (gross-net) and net N_2_O production by gut extracts from the eleven termite species.

Parameters	R^2^_adj_(%)	Pmod	Preditor variables	P-value	Contribution of variable to the model (%)
gross N20	92.1	***	Total -nir	***	56.6
			AOB	***	28.2
			Total-com	***	11.9
N20 reduction	78.4	***	*nosZ1/nirk *	***	64.7
			*nosZ1*	**	23.9
			*nosZ2*	*	11.4
net N20	81.6	***	*nosZ1/nirk*	***	48.8
			*nosZ1*	**	44.1
			*nosZ2*	**	7.1

The second and third columns give the adjusted R2 and p values of the models. The last columns give the selected predictors(microbial abundances and abundance ratios) and their p values and relative contribution to the models. The p values are indicated as *0.01<p<0.05; **0.01<p<0.001; ***p<0.001.

## Discussion

### Both gross and net N_2_O production rates from termite gut vary greatly between species, with particularly low rates for wood feeders

For the first time, we quantified the potential denitrification rates by the gut of termite species from different feeding guilds to determine whether the observed differences in net production rates are due to different gross production rates and/or different consumption-to-production ratios. Our results are broadly consistent with those of a previous study (Brauman et al., 2015), which found that three fungus-growing and four soil-feeding termite species had high net emission rates and, four wood-feeding termite species had weak N_2_O sinks. These authors suggested that wood-feeders do not produce, or produce very little, N_2_O because of the N deficiency in their diet (although termites in this guild have N_2_-fixing bacteria in their gut that compensate for the lack of N in their diet (Breznak et al., 1973; Benemann, 1973). In contrast, fungus-growing and soil-feeding termites are a source of N_2_O, most likely due to the high N concentration in the termite gut (Brauman et al., 2015). For example, fungus-growing termites acquire N through their fungal symbiont, *Termitomyces*, which is a rich source of N (Rouland-Lefèvre, 2000). Soil-feeding termites can take benefit from N sources like peptides and other nitrogenous soil compounds (Ji et al., 2000; Ji and Brune, 2006). Our results for the grass-feeding termite species *Trinervitermes* sp. (high N_2_O production) contradicted previous findings (Brauman et al., 2015), which reported low or even negative net N_2_O emission by this species. However, these authors reported that N_2_O emission rates from grass-feeding termites varied greatly, ranging from minor N_2_O consumption for some species to *P. spiniger* and *H. mossambicus* production rates comparable to soil feeders. Furthermore, high levels of nitrate (NO_3_^-^) and ammonium (NH_4_^+^) were found in the gut of *Trinervitermes* sp (Brauman et al., 2015), as would be expected for termites feeding on N-rich material. The net N_2_O production rates observed here suggest that grass-feeding termites do produce N_2_O. Overall, our results support the hypothesis that termites’ diet is a key determinant of their N_2_O production rates. The same conclusion has been drawn for earthworms (Depkat-Jakob et al., 2010), one of the significant N_2_O producers among soil invertebrates. It has been reported that the invertebrate fauna has an effect on N_2_O emissions from soils (Kuiper et al., 2013). However, the ability of soil invertebrates to reduce N_2_O to N_2_ has only been demonstrated for a few species of soil-feeding termites (Ngugi et al., 2011) and earthworms (Wüst et al., 2009; Peter et al., 2013).

For the first time, we assessed to which extent potential gross (N_2_O + N_2_) and net N_2_O production rates varied across termite species from five feeding guilds. The potential gross N_2_O rates measured in soil-feeding termite guts varied but remained within the same range as the previously studied species (Ngugi et al., 2011). Notably, we found that the potential gross N_2_O production rates from termite gut homogenates of *Macrotermes muelleri* (fungus-growing termite), *Cubitermes speciosus* and *Crenetermes albotarsalis* (two soil-feeding species), and *Trinervitermes* sp. (grass-feeding) were significantly higher than potential gross and net N_2_O production rates for wood-feeding termite species. Furthermore, net production rates were always significantly lower than gross production rates. Our results suggest that the reduction of N_2_O to N_2_ is a general trait in termite guts regardless of feeding guild. Despite the fact that approximately 90% of the N_2_O produced was consumed, the effects of termite species on N_2_O production were roughly consistent when net or gross production rates were considered (e.g., 15-20 times lower gross production rates and 5-30 times lower net production rates for wood-feeding species than other species). The overall correlation between the potential net and gross N_2_O production rates is consistent with reports of significant correlations between gross and net N_2_O production rates from soil (Florio et al., 2021), although this is not always the case (Assémien et al., 2019).

For a subset of five termite species, we also compared N_2_O production from live termite individuals (per g termite dry mass) with potential net production by gut homogenates (per g gut dry mass). Given that the weight of a whole termite is primarily made up of its gut/gut content, this comparison is sound. The actual N_2_O production rates from live termites were 1.5 to 3 times lower than the potential net N_2_O production rates by termite gut, most likely because the potential net production assays were performed under optimal conditions (i.e., the surplus of nitrate and electron donors provided to the termite gut microbiota and strict anoxic conditions prescribed). Furthermore, we found consistency between the N_2_O production from live termite individuals and potential net production by gut homogenates for the subset of five termite species tested, confirming –for these species– the hypothesis that N_2_O production by live termites primarily reflects the balance of N_2_O production and N_2_O consumption in the termite gut. This demonstrates the importance of studying the termite gut microbiome in order to understand their N_2_O emissions.

#### The N cycle-related microbiome of the termite gut differs strongly between termite species, with wood feeders having particular abundance profiles of nitrifier and denitrifier groups

The abundance of each functional gene varied considerably depending on termite species, with ammonia oxidizers having a 100 to 10,000-fold range and comammox and denitrifiers having an even wider range. Our results are consistent with the significant differences in functional gene abundances between live termite species as previously reported (Brauman et al., 2015). Furthermore, the detection of comammox (from clade A and clade B) and *nosZ2*. N_2_O reducers in the gut of the termite species studied (which had never been tested before) supports the hypothesis that these microorganisms are widely distributed in the environment (Hallin et al., 2018).

The presence of a specific N cycle-related microbiome in termite species that feed on soil and wood is suggested by differences in functional gene abundances among termite species. The gut microbiota of the wood and soil/wood-feeding termites were characterized by lower abundances of *nirK* nitrite reducers and *nosZ1* N_2_O reducers, but higher abundances of all nitrifier groups, including comammox (though the trend was not significant for AOB). We initially thought that nitrifiers could explain the low N_2_O production rates by wood-feeding termites because (i) ammonia oxidation is often thought to be the limiting step of nitrification and could control the nitrate supply to denitrifiers, and (ii) nitrifiers could produce N_2_O as a by-product with particularly high N_2_O yield for AOB (Prosser et al., 2020). However, our results do not suggest that nitrifiers play a significant role in explaining the low N_2_O emission rates of wood-feeding termite species because all nitrifier groups were abundant in wood-feeding termite guts, regardless of termite taxonomy classification (higher or lower termites). More specifically, the significantly higher nosZ/nir ratio observed for lower termite species suggests that the microbiomes of the higher and lower termites within the wood-feeding guild differ. The microbiota in the hindgut of lower termites is already known to be dominated by flagellated protists, whereas higher termites have only a small number of gut protists (Hongoh, 2011). The high nosZ/nir ratios observed in wood-feeders were in contrast to those observed in soil and grass-feeding termite species, with the latter being in the same range as values reported for earthworm values (Majeed et al., 2013).

Our results point to possible niche differentiation within two N cycle-related microbial functional groups: the first between *nirK-* and *nirS-*harboring bacteria within the nitrite reducer group, and the second between *nosZ1-* and *nosZ2-*harboring bacteria within the N_2_O reducers. Previous studies have a suggested that these groups may have niche differentiation. For example, in a study of soil denitrifiers in an African savanna (Assémien et al., 2019) demonstrated that *nirK* and *nirS* bacteria responded to different soil environmental conditions, as did *nosZ1* and *nosZ2* N_2_O reducers. In particular, these groups responded differently to nitrate levels, organic carbon availability, and pH in grassland soils. The abundance of *nirK* was positively related to soil nitrate and negatively related to soil C, whereas the reverse was true for *nirS* abundance (Xie et al., 2014). A meta-analysis revealed that *nirS* and *nirK* bacteria respond differently to environmental gradients (Jones and Hallin, 2010). Similarly, several studies have suggested that *nosZ1* and *nosZ2* N_2_O reducers may have different niches in a variety of environments, including soils (Orellana et al., 2014; Assémien et al., 2019), wetlands (Ligi et al., 2015; Saarenheimo et al., 2015), and coastal sediments (Wittorf et al., 2016). According to genome analyses and culture-based studies, the physiology of N_2_O reduction by *nosZ* bacteria differs between clade 1 and clade 2 (Hallin et al., 2018). This niche differentiation among nitrite reducers and N_2_O reducers may explain why the N-related microbiome of wood-feeding termite guts differs from that of other termite species. However, functional diversity within each microbial group can still be high, particularly in *nirK* and *nirS* bacteria (Xie et al., 2014). The nitrifier, and denitrifier taxa that are better adapted to the gut conditions of wood-feeding termites than in the other guilds will require in-depth characterization.

### Denitrifier and nitrifier abundances in termite gut are good predictors of gross and net N_2_O production rates

So far, no relationship has been found between the abundances of gut nitrifier and denitrifier groups and gross and net N_2_O production rates by live termites or termite gut. Nevertheless, in many ecosystems, the abundance of nitrifying and denitrifying genes has often been used as a predictor of N_2_O production and reduction (Philippot, 2006; Morales et al., 2010; Petersen et al., 2012). Our results show that the key driver of potential gross N_2_O production from termite gut was the total abundance of nitrite reducers involved in the first step of denitrification, in which NO_2_^-^ is reduced to NO (and then to N_2_O because NO can be toxic within cells). AOB, and comammox abundances also played a role, albeit to a lesser extent. This suggests that denitrification is important in termite N_2_O production. Nitrifiers, on the other hand, could play a role either directly by producing N_2_O or indirectly by supplying N to denitrifiers (influencing the activity of denitrifier cells). Previous soil research has found either a significant relationship between potential gross N_2_O production and the abundances of *nirS* and/or *nirK* nitrite reducers (with 29–91% of the variance explained (Dong et al., 2009; Enwall et al., 2010; Henderson et al., 2010; Attard et al., 2011; Petersen et al., 2012; Assémien et al., 2019) or a lack of relationship (Philippot et al., 2009; Le Roux et al., 2013). This is due to the fact that denitrification is a facultative process (Zumft, 1997), and thus potential denitrification (also known as denitrification enzyme activity) is not always proportional to denitrifier abundance. In contrast, nitrifier abundance has often been reported to be more closely related to potential or actual nitrification rates (Le Roux et al., 2013), although changes in environmental conditions can obscure these relationships.

Our results also show that the abundances of *nosZ1* N_2_O reducers and *nirK* nitrite reducers, as well as the ratio between these gene abundances, driven both potential N_2_O production and potential N_2_O consumption rates in termite guts, with these variables explaining 82% of the variance in the potential net N_2_O production. This suggests that *nirK* nitrite reducers and *nosZ1* N_2_O reducers play an important role in N_2_O production and consumption in termites. This is consistent with a recent study (Assémien et al., 2019) that found net N_2_O production by savanna soils was correlated with both gross N_2_O production and *nosZ1* abundance, but not with *nosZ2* abundance (though *nosZ2* bacteria were more abundant than *nosZ1* bacteria in these soils as in the termite guts we studied). N_2_O emissions from semi-arid grassland soils were also related to *nirK* abundance but not *nirS* abundance (Shi et al., 2021).

Overall, our results show that understanding the abundances of N-cycling functional genes can help explain why gross and net N_2_O production rates vary so much between termite species, with *nirK* nitrite reducers and *nosZ1* N_2_O reducers likely playing a major role. Therefore, future studies should prioritize analyzing the diversity of *nirK* and *nosZ1* bacteria in termite guts to better understand the variation in N_2_O emission rates between termite species and the low N_2_O emission rates observed for wood-feeding termites. Furthermore, it has been demonstrated that the physical and chemical conditions prevailing in termite guts vary greatly depending on the gut compartment. Denitrification, for example, was restricted to the posterior hindgut in homogenates of individual gut sections. In the anterior gut, however, dissimilatory nitrate reduction to ammonium (DNRA) was the dominant process (Ngugi and Brune, 2012). Although small amounts of N_2_O are produced during DNRA (Gödde and Conrad, 1999), our goal was not to differentiate N_2_O sources in each gut section but to assess variations in net and gross N_2_O production in the entire intestinal gut from various termite species. Future research aimed at distinguishing termite gut compartments could help to understand the role of compartmentalization in the simultaneous functioning of the various N-cycling microorganisms in termite guts.

## Data availability statement

The original contributions presented in the study are included in the article/**Supplementary Material**. Further inquiries can be directed to the corresponding author.

## Author contributions

EM and XLR designed the study, discussed the results, and wrote the manuscript; TJ performed N_2_O emission experiments, analyzed the data, and contributed to the manuscript, AR identified the termites, contributed to the setting up of experiments; CDC and AD performed and analyzed qRT-PCR data of N-cycle marker genes. All authors read and approved the final manuscript.

## Funding

This work was funded by the Institute of Ecology and Environmental Sciences of Paris (iEES) and by the National Research Institute for Agriculture, Food and Environment INRAE (ECODIVDepartment) via the Microbial Ecology laboratory (UMR 1418 INRAE).

## Acknowledgments

The authors thank Dr. Corinne Rouland-Lefèvre for granting access to the termite rearing room at IRD Bondy France Nord.

## Conflict of interest

The authors declare that the research was conducted in the absence of any commercial or financial relationships that could be construed as a potential conflict of interest.

## Publisher’s note

All claims expressed in this article are solely those of the authors and do not necessarily represent those of their affiliated organizations, or those of the publisher, the editors and the reviewers. Any product that may be evaluated in this article, or claim that may be made by its manufacturer, is not guaranteed or endorsed by the publisher.
