# Supplementary material for: Potential gross and net N2O production by the gut of different termite species are related to the abundance of nitrifier and denitrifier groups
Source: Front Microbiomes. 2022 Oct 18;1:1017006. doi: 10.3389/frmbi.2022.1017006 (PMC12993464; doi:10.3389/frmbi.2022.1017006)
Supplement: Supplementary file 1 [file DataSheet_1.docx]

**Supplementary materials**

**Supplementary Table 1.** Termite species and taxonomic affiliation


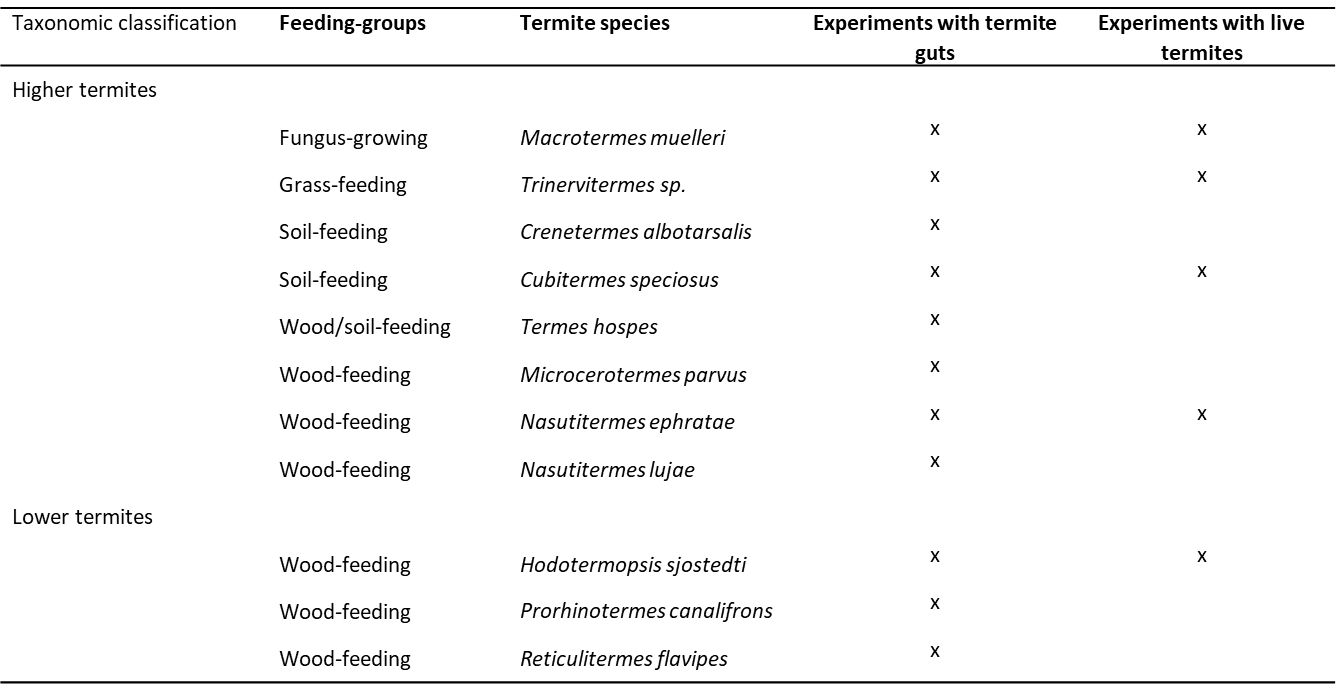


**Supplementary Table 2.** Primers and conditions of RT-PCR for each N-cycle functional marker genes

**
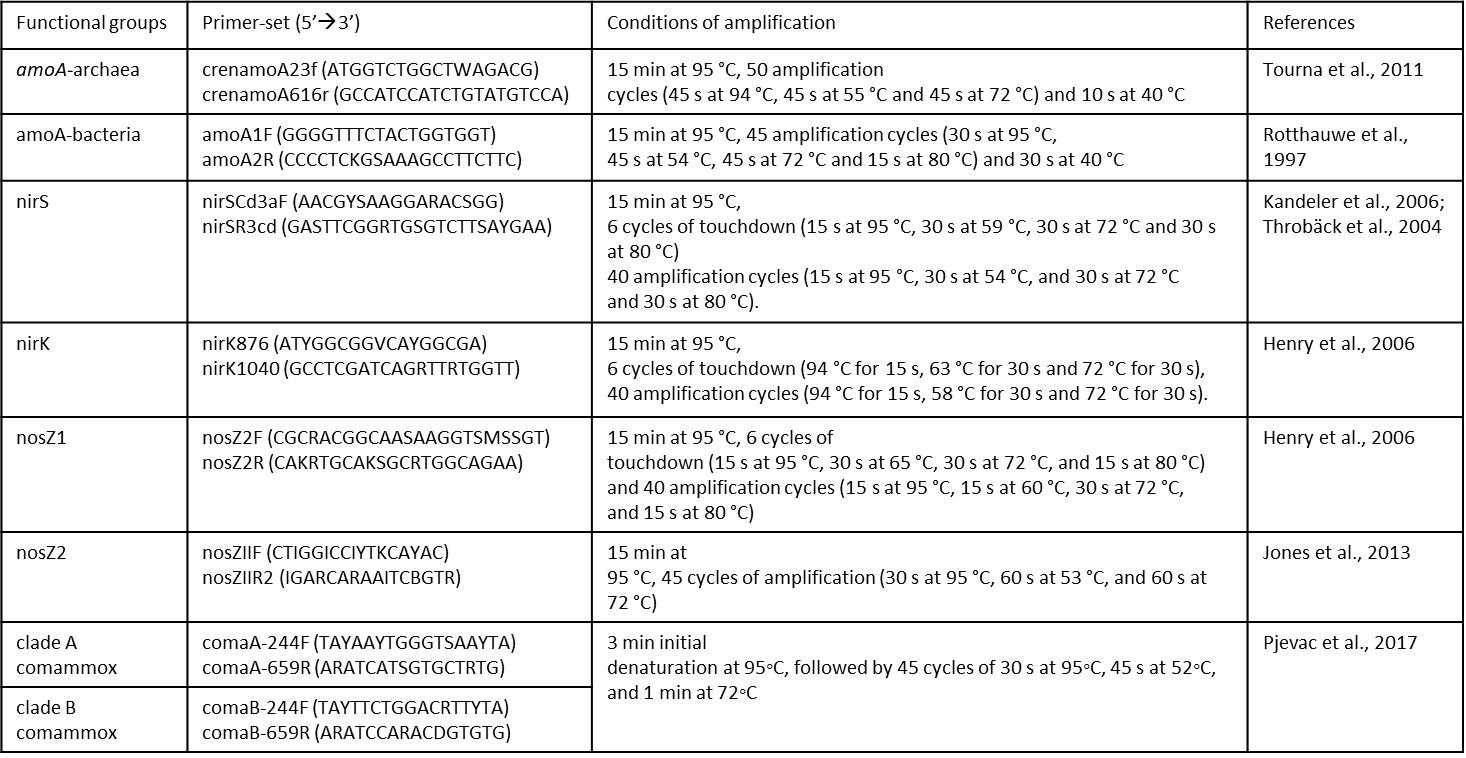
**

**
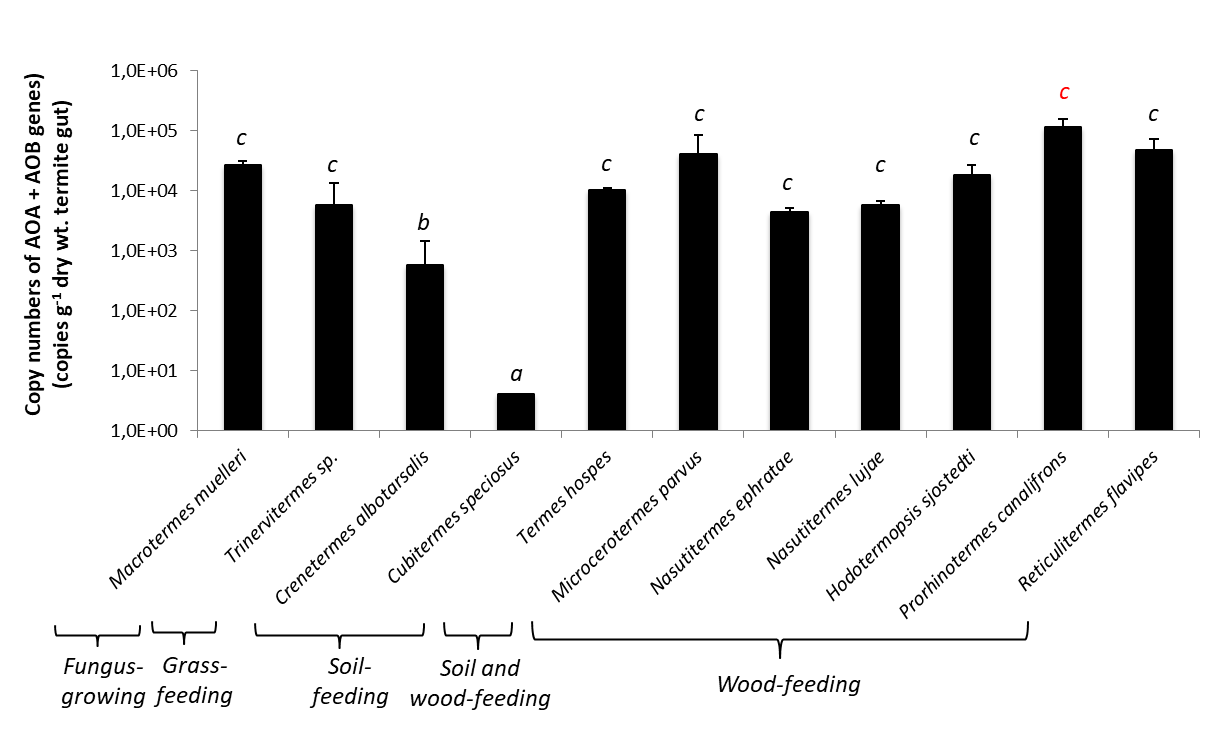
**

# Supplementary Figure 1. Total abundance of ammonia oxidizers, *amo-*AOA and *amo*-AOB gene copy numbers in the gut of the 11 termite species. Data represent mean (n=5) and standard deviation (error bar). Different letters above bars indicate a significant difference (*P* < 0.05) among species.

**
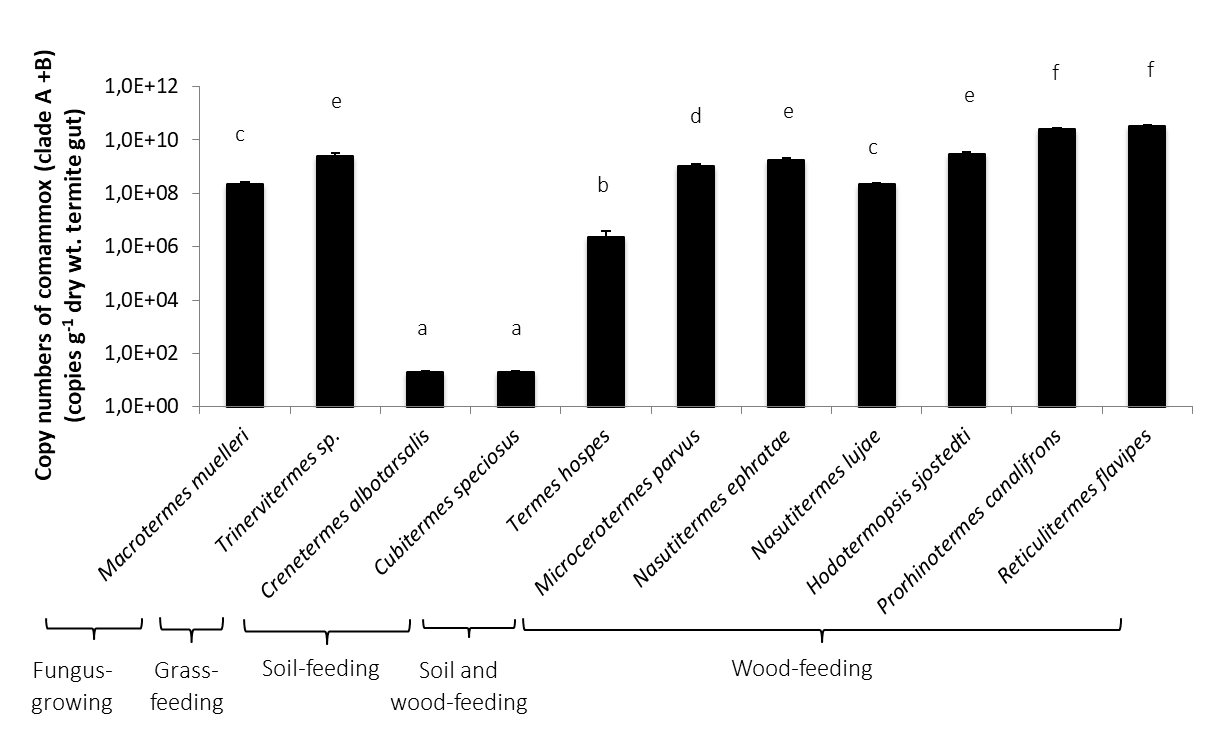
**

# Supplementary Figure 2. Total abundance of comammox from clade A and clade B in the gut of the 11 termite species. Data represent mean (n=5) and standard deviation (error bar). Different letters above bars indicate a significant difference (*P* < 0.05) among species.

**
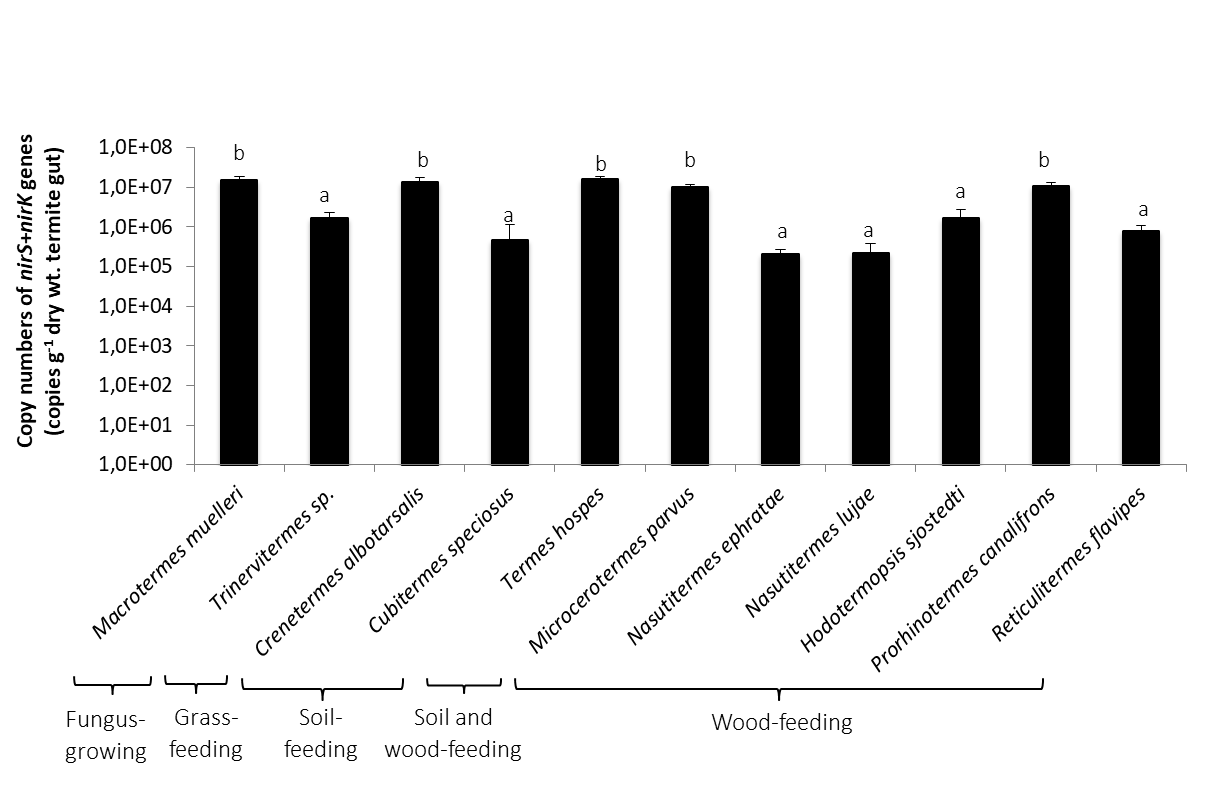
**

# Supplementary Figure 3. Total abundance of nitrite reducers, *nirK* and *nirS,* in the gut of the 11 termite species. Data represent mean (n=5) and standard deviation (error bar). Different letters above bars indicate a significant difference (*P* < 0.05) among species.

**
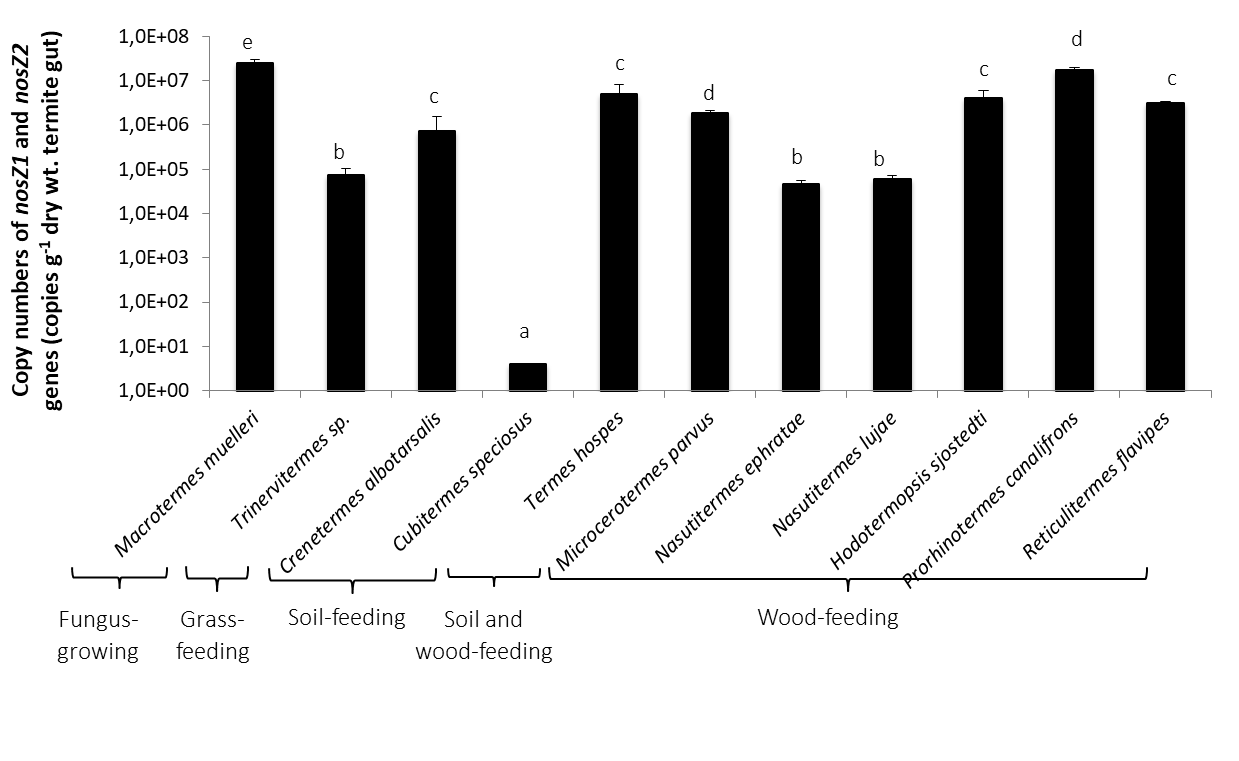
**

# Supplementary Figure 4. Total abundance of N_2_O reducers, *nosZ1* and *nosZ2*, in the gut of the 11 termite species. Data represent mean (n=5) and standard deviation (error bar). Different letters above bars indicate a significant difference (P < 0.05) among species

**Supplementary Table** **3.** Results of one-way ANOVA testing whether N-functional gene abundances and ratio of N-functional gene abundances differed among lower and higher termites. ComA and comB refer to complete ammonia oxidizers from clades A and B, respectively; nirK and nirS refer to nitrite reducers harboring the *nirK* and *nirS* genes, respectively; *nosZ*1 and *nosZ*2 refer to N_2_O reducers with nosZ1 and *nosZ*2 genes, respectively; AOA and AOB refer to archaeal and bacterial ammonia oxidizers, respectively; nir, nos, amo and com correspond to the total abundances of nitrite reducers, N_2_O reducers, ammonia oxidizers, and complete ammonia oxidizers, respectively. F values and significance level: ns: not significant; ^⁎^p < 0.05; ^⁎⁎^p < 0.01; ^⁎⁎⁎^p < 0.001.

|  | | *comA* | | *comB* | | *nirK* | | *nirS* | | Nir | *nosZ1* | | *nosZ2* | | *nos* |
| --- | --- | --- | --- | --- | --- | --- | --- | --- | --- | --- | --- | --- | --- | --- | --- |
| F value | | 57.9 | | 15.5 | | 1.9 | | 0.07 | | 1.07 | 0.94 | | 23.6 | | 2.7 |
| P | | 0.0000*** | | 0.0003*** | | 0.17^ns^ | | 0.79^ns^ | | 0.31^ns^ | 0.34^ns^ | | 0.0000*** | | 0.11^ns^ |
|  | AOA | | AOB | | *amo* | | *amo*/*nir* | | *amo*+*com*/*nir* | | | *nos*/*nir* | |  |  |
| F value | 4.2 | | 42.2 | | 7.1 | | 6.9 | | 11.3 | | | 69.6 | |  |  |
| P | 0.04* | | 0.0000*** | | 0.01* | | 0.01* | | 0.002** | | | 0.000*** | |  |  |
